# Risk Assessment Matrices for Workplace Hazards: Design for Usability

**DOI:** 10.3390/ijerph19052763

**Published:** 2022-02-27

**Authors:** Roger C. Jensen, Royce L. Bird, Blake W. Nichols

**Affiliations:** Safety, Health, and Industrial Hygiene Department, Montana Technological University, Butte, MT 59701, USA; rbird1@mtech.edu (R.L.B.); bnichols@mtech.edu (B.W.N.)

**Keywords:** occupational safety, risk assessment, system safety, risk, usability, risk terminology, survey, severity terms

## Abstract

In occupational safety and health (OSH), the process of assessing risks of identified hazards considers both the (i) foreseeable events and exposures that can cause harm and (ii) the likelihood or probability of occurrence. To account for both, a table format known as a risk assessment matrix uses rows and columns for ordered categories of the foreseeable severity of harm and likelihood/probability of that occurrence. The cells within the table indicate level of risk. Each category has a text description separate from the matrix as well as a word or phrase heading each row and column. Ideally, these header terms will help the risk assessment team distinguish among the categories. A previous project provided recommended sets of header terms for common matrices based on findings from a survey of undergraduate OSH students. This paper provides background on risk assessment matrices, discusses usability issues, and presents findings from a survey of people with OSH-related experience. The aim of the survey was to confirm or improve the prior recommended sets of terms. The prior recommendations for severity, likelihood, and extent of exposure were confirmed with minor modifications. Improvements in the probability terms were recommended.

## 1. Introduction

### 1.1. Background on Risk Assessment

The practice of occupational safety and health (OSH) has undergone a 50-year transition from being a mostly rule-following practice into a multi-faceted profession blending rules and risk management processes to achieve effective and feasible protection for employees, property, environment, and other business interests [1,2,3]. Risk management today involves several processes, repeated periodically, to identify hazards, evaluate the associated risks, and assess various tactics for preventing and mitigating harm from those risks [2,3,4]. A tool used for assessing and evaluating risks is referred to in the OSH field as a risk table, risk grid, risk matrix, or (our preference) risk assessment matrix (RAM) [2,3,5,6,7,8,9,10,11].

RAMs appear as a two-dimensional grid with one axis having categories of harmful consequence and the other axis with categories for likelihood or probability. The cells inside the grid are used to indicate risk. Risk-assessment teams use RAMs as part of an organization-specific risk management process [2,3,5,7,8,11]. Although the details differ somewhat, a risk-management process involves: (1) identifying hazards and the associated risks, (2) determining tactics for reducing/mitigating each risk, also called risk treatment, (3) assessing the risks in terms of credible harmful consequences and likelihood of occurring, (4) evaluating each hazard-specific risk in terms of the organization’s tolerance for risk, (5) communicating with those affected, (6) implementing the approved risk-reduction tactics, and (7) following up by monitoring implementation and effectiveness. RAMs are tools used in Process 3 (risk assessment) and Process 4 (risk evaluation).

A RAM can be used in Process 3 to analyze risks of a specific hazard, document effect from each risk-reduction tactic, and provide useful information for Process 4. This involves following steps that can later be used to document having used due diligence or reasonable care (depending on the applicable legal system). The hazard-specific assessment process described by Jensen [2] begins by using a RAM to establish a baseline risk by assuming the hazard has not yet incorporated any attempt to prevent or mitigate the harm. It involves judging the consequence of one or more foreseeable harmful event and the likelihood of occurrence. For each risk-reduction tactic added, the RAM is used to document the effect of that tactic by reducing severity or likelihood. This process is performed again and again, each time an additional risk-reduction tactic is considered, thereby, providing a documented trail of having taken safety seriously [2]. Thus, an organization’s RAM serves as a core tool for use by risk-assessment teams to characterize risk in a systematic manner. Completed RAMs provide information in a visual format for Process 4 involving the evaluation of the risks and deciding if the organization can tolerate the remaining risks [2,3,5,6,8,9,10,11].

This paper provides background on the numerous variations in RAM designs, the means for characterizing level of risk, and options for helping the individuals who use RAMs to achieve reasonable accuracy and precision. A typical use of a RAM is to have a small team use it as a tool for assessing various hazards. In OSH, the people who serve on risk-assessment teams have varying backgrounds in education, experience with the types of hazards being assessed, and experience applying RAMs. Thus, in selecting an appropriate RAM for use by an organization involves recognizing that a RAM is a tool for use by people and should, therefore, be designed for human usability. At the very least, a RAM should be designed for usability by engineers, operations personnel, and others likely to be assigned to risk-assessment teams.

**Figure 1:**
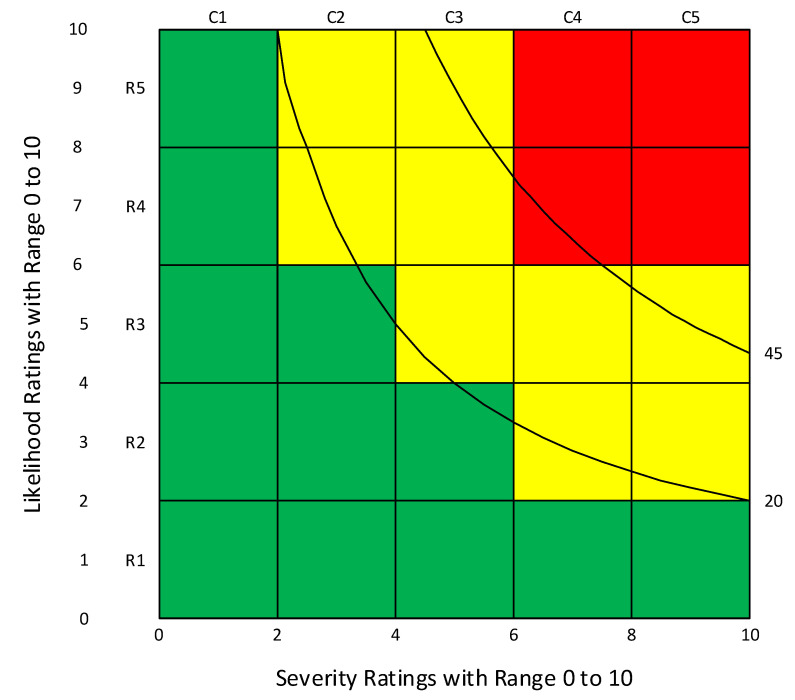
One of many possible designs of a risk assessment matrix. It uses five rows and five columns with three color-coded bands for cells with similar risk levels. Both axes were normalized to the range 0–10. The two iso-risk lines indicate risks from Row × Column = 20 and 45.

The substantial body of literature about RAMs reflects articles based on reasoning, experience, and expert opinion [8,9,10,11,12,13,14,15,16,17,18]. Few papers on RAMs report empirical research. The authors of this paper have identified four empirical studies on RAMs. Two studies examined how health service providers conduct risk management [19,20]. Card, Ward, and Clarkson reported a content analysis of health services organizations in the East of England area of the British National Health Service. They found the risk management systems were weak in two main areas: (i) guidance to support risk evaluation methods, including use of a RAM, and (ii) organizational guidance to support risk control [19]. In a second empirical study, Kaya, Ward, and Clarkson sent requests to 160 hospitals in England for descriptions of the RAMs they use [20]. Out of 100 responses, 99 used a 5-row by 5-column matrix similar to the one in Figure 1. The 99 RAMs used the order number of rows and order of columns to fill the cells in the matrix with numbers obtained by multiplying the applicable order numbers. These numerals were used to sort cells with similar risk into bands identified by a particular color. In the study, each cell had a number ranging from one to 25; however, the healthcare providers differed in how cells were assigned to the colored levels of similar risk. This resulted in 28 different RAMs. The 99 hospitals used three, four, or five colored risk bands in their matrices [20]. The number of bands and number of hospitals were as follows: three bands (23), four bands (70), and five bands (6).

In a third empirical study, Ball and Watt reported a campus study of using a 5 × 5 RAM to assign a risk score to three photos of public places with unprotected edges where deadly falls could occur [12]. Their students had received basic instruction on the use of a RAM, but no specific training on how to judge likelihood or severity [12]. They found students had poor accuracy and precision. In a fourth study, Jensen and Hansen surveyed undergraduates studying OSH to determine how they understand various words and phrases used in RAMs [21]. Using results, the researchers identified sets of terms most suitable for naming the row and column categories in RAMs [21]. This article provides background on RAMs followed by a description of this follow-on survey of individuals with at least two years of OSH-related experience undertaken with the aim to reexamine the prior recommended word sets to determine if the prior recommendations are confirmed, or if improvements are desirable.

### 1.2. Diverse Options for Design

Organizations may design and use a RAM of their choosing. This has the advantage of allowing organizations to match their needs and values. There are, however, many RAMs that contain inherent pitfalls, inconsistencies, and difficulties in usability [8,9,10,11,12,13,14,15,16]. To explain the various ways that RAMs can differ, some terms need clarification. Figure 1 serves as a point of reference RAMs come in different sizes, commonly described by the number of rows and number of columns. The size of the example in Figure 1 is a 5 × 5. The size of a RAM affects the resolution—more categories mean greater resolution. While it appears desirable to have large resolution, the RAM designer should recognize that assigning categories for likelihood and severity is a subjective process that is not well suited for making fine distinctions between adjacent categories [8,12]. Therefore, as Baybutt advises, the number of levels “should be consistent with the ability of practitioners to discriminate between levels” [8].

**Figure 2:**
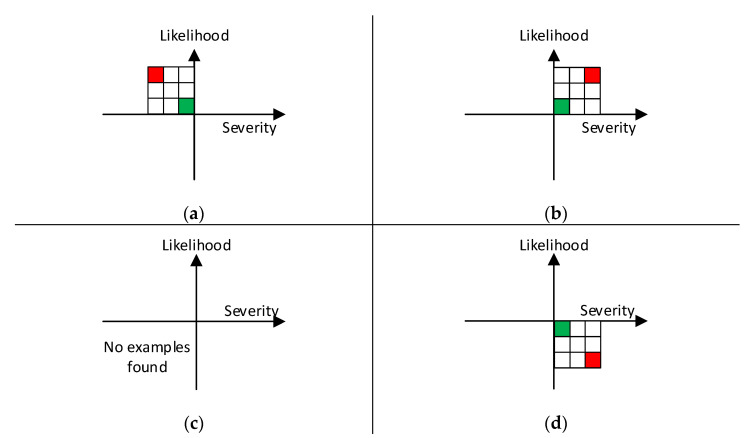
Positive and negative axes of RAMs presented in quadrants of Cartesian coordinate system. Panels (**a**–**d**) depict quadrants II, I, III, and IV, respectively.

**Figure 3:**
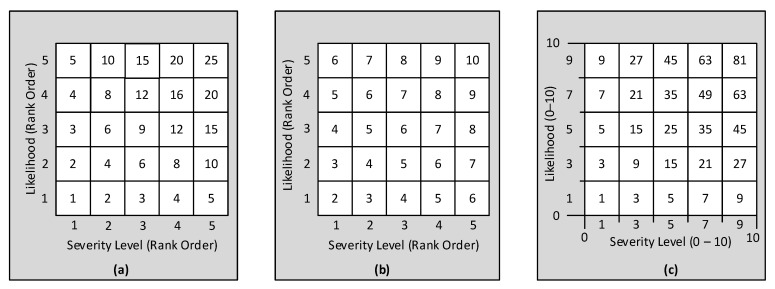
Examples of three distinct methods for assigning risk indicators to the cells of risk matrices. Panels are: (**a**) multiplication of order numbers, (**b**) addition of order numbers, (**c**) multiplication of the midpoint values of the applicable row and column categories.

RAMs are presented in different orientations. Figure 2 depicts possible orientations of a 3 × 3 RAM using the Cartesian coordinate system to establish the positive and negative directions of rows and columns. In each RAM, the green colored cell is the lowest risk; the red cell is the greatest risk. Panel a depicts a RAM in quadrant II. This is illustrated by MIL-STD-882E [22] and others [11,14,22]. This quadrant fits activities for which the horizontal axis applies to expected loss; the business community assigns a negative value to losses. Figure 3b depicts a RAM in quadrant I. That is the location of RAMs emphasized in this paper and others [6,10,12,13,16,17,18,19]. Figure 3c is a location where both axes are negative. The authors did not find any examples of a RAM located in quadrant III. Figure 3d depicts a RAM in quadrant IV. Three examples have been found [7,8,23].

The columns in Figure 1 are for amount of harm—commonly called severity or consequence. Severity and consequences may relate to either financial loss or harm to personnel or other. For OSH practice, the term severity is most conventional and is used throughout this paper. Columns are for distinguishing ordered categories of severity

A RAM needs a key containing a text description of each severity category to explain and illustrate what makes each column different from adjacent columns. Another essential attribute of the severity categories is that they must be put in order such that each is clearly greater than the next lower category [8,11,13,15]. In addition to the text description, each column has a header term at the top. In Figure 1, the five column headers are indicated by variables C1, C2, C3, C4, and C5. The project described in this paper explored various terms for these column headers.

The rows in Figure 1 are for the ordered categories of how likely the hazardous event or exposure will occur. Four ways to describe the row categories were used in this paper. Probability was used for quantitative ratings with values in the range 0.0–1.0 or a multiple of 10. Likelihood refers to qualitative judgments expressed numerically or nominally (without numbers). A third dimension included in the present study is extent of exposure, a term that includes measures used to account for employees very rarely exposed to a hazard versus employees regularly exposed to the hazard. Extent of exposure is expressed by the frequency or duration of employee exposures to the hazard per a specific unit of time, e.g., three times per year, three exposure-hours per week, 80 uses per month. Extent of exposure may be used as a third dimension of a RAM or may be incorporated within the rows of a 2-dimensional RAM by inclusion in the descriptions provided in the key. A dimension not studied in this survey is frequency; it is used in the process industries to distinguish rows categories in a RAM. Common uses include 1 death/10 years, 1 death per 100 years, and 1 death per thousand years. This project addressed sets of terms to replace the generic row headers in Figure 1 (R1, R2, R3, R4, and R5).

For a specified hazard, the individuals participating in a risk assessment are expected to both foresee possible hazard scenarios and estimate how likely each may occur [5,6,7,8,9,10,11,12,13,14,15,16,17]. These projections must then be put into the column and row categories of the applicable RAM. Two aids for helping risk assessment team members select column and row categories that match their projections are, first, explicit descriptions in the RAM’s key, and second, the terms used to label each column and row category. The authors developed this project with intent to help RAM designers with the second of these aids—selecting sets of terms for both column and row headers.

The cells in a RAM indicate level or risk. Colors are often used to show groups of cells with similar risk levels, known as risk bands. In Figure 1, red cells denote the highest risk band and green cells denote the lowest risk band. Yellow cells are those separating green and red cells. For OSH, a hazard rated in the green band is generally considered tolerable or acceptable, and a hazard in the red band is typically considered highly undesirable or not tolerable [5,6,7,8,9,10]. While the decisions associated with red and green cells are often stated as clear-cut rules, the preferred practice is to consider these as indicators to assist with making decisions [8,9,10,11,12,13,14,24]. Cells rated in the yellow band indicate a need for additional attention in order to reduce the risk to as low as reasonably practicable (ALARP) prior to deciding on tolerability. After achieving ALARP, the organization’s risk-assessment team uses the final RAM as a visual tool to communicate with the organization’s decision makers about tolerability [18].

The basic definition of risk in Equation (1) provides the basis for using a table format [2,3,6,8,9,10,11,13]. According to Equation (1), the probability of a harmful event B occurring (*P_B_*) is multiplied by expected loss, given that B occurred.

Risk = *P*_B_ × (Loss|B) (1)

A risk assessment matrix provides an easily understood depiction of risk being based on the product of applicable values in the row (probability or likelihood) and column (severity). Although this approach has been a tradition in the field of system safety, the OSH community has, for various reasons, sought a less quantitative approach [5,7,8,9,10,11,15,19,20].

The risk matrices in Figure 3 illustrate three ways to express risk within the cells. Each matrix uses rows for likelihood and columns for severity. In Figure 3a,b, the rows are numbered 1–5 in order from lowest to highest likelihood, and the columns are numbered 1–5 in order from least to greatest severity of harm. With that start, there are two ways to assign numerical risk indicators (RI_ij_) to the cells. Using the notation that subscripts i and j refers to row and column, respectively, R refers to rows, and C refers to columns, one method is to determine the RI values in cells is RI_ij_ = R_i_ × C_j_. That yields the values in the Figure 3a matrix. The other method is to add the values using RI_ij_ = R_i_ + C_j_. That yields the values in the Figure 3b matrix [6,11]. The approach in Figure 3a assumes the category-to-category increases are basically linear. The approach in Figure 3b assumes the categories in both the rows and columns are spaced logarithmically so that each category is approximately 10 times greater than the next lower category [6,10,11].

The third approach to quantify a risk matrix is to take the established row and column values, normalize each to a common scale (e.g., 0–1, 0–10, or 0–100), and use the normalized row and column matrix for establishing a less complex RAM, for which Figure 3c is an example. The row and the column categories are then defined in terms of those values. In the Figure 3c example, a 5 × 5 matrix may have a 10-point axis divided so that five equal width categories have upper bounds at 2, 4, 6, 8 and 10. The risk indicators in each cell are the product of the mid-range value of the respective row category (1, 3, 5, 7, 9) and the mid-range value of the respective column (1, 3, 5, 7, 9). This mid-point approach corresponds to instructing a RAM assessment team to assign severity categories based on the most representative sort of harm the team members can foresee, and likelihood categories based on the reasonably foreseeable chance of occurrence.

Several insightful papers have been positive on the approach of using the framework depicted in Figure 3c [8,11,12,13,17,19,20]. These authors of these papers expressly recognize the approach as being a simplified version of an underlying quantitative matrix. Mathematical justification for the approaches in Figure 3b and Figure 3c have been provided by Rausand [6] (pp. 102–103) and Cox [13], respectively.

The next challenge is to determine how to distinguish the cells for highest risk (colored red) from cells with lower risks (colored green). One approach is to follow the axioms developed by Cox [13]; the other approach is to use the iso-risk contour-based method [14,24]. The RAM in Figure 1 was created using the iso-risk contour method by which green cells were located below or left of the iso-risk line 20, and red cells were located above and right of the iso-risk line 45. For cells bifurcated by an iso-risk line, color was assigned based on the side of the line with the largest area of the cell.

Referring to the RAM in Figure 1, the cells colored green have risk values per Equation (1) in the range 0–24, while the red cells have risk values in the range 36–100. The red-color band includes the upper right cell plus three adjacent cells. All cells not colored green or red are assigned the color yellow.

Breaking each axis into categories defined as portions of the full range helps with usability by the risk-assessment teams, first, by not asking assessors to understand the underlying mathematics, and, second, by not expecting them to spend countless hours discussing the precise number to use for each row and column value. Discussions of RAMS frequently include a distinction between qualitative and quantitative forms. A quantitative RAM, for example, has probability values for the row categories, monetary values for the columns, and the cells values are computed with Equation (1) resulting in risk values in monetary units. Qualitative RAMs have rows and columns defined nominally and cells assigned risk categories such as high, medium, and low [2,17]. Cox, Babayev, and Huber [17] provide examples of regulatory agencies that use this approach. A third form of RAM, often called semi-quantitative, has each axis divided into ordered categories and assigned numerical values based on their order. Figure 3a,b are examples. A fourth type of RAM, illustrated in Figure 1 and Figure 3c, consists of (i) both axes using linear scaling and the same range (e.g., 0–10), and (ii) risk indicated by the product of the respective row and column values. Appendix A provides a conceptual explanation of how this fourth type of RAM can approximate an underlying quantitative relationship based on Equation (1).

The domain of application may, or may not, warrant different matrices. Employers using, or planning to adopt, a RAM need to ponder some things about the hazards involved [8,11]. In what kind of industry will the RAM be used? For what types of hazards will the RAM be used as a tool for risk assessment? Related to this issue is the temptation to have one RAM for all applications in the organization. This approach has been criticized by multiple authors who recommend different RAMs for different consequences, e.g., employee safety, property damage, environmental harm, business interruption, or community relations [9,14,15]. Baybutt [10] recognized the pitfalls of using one matrix for diverse domains and proposed a method for calibrating the matrix for different domains within an organization.

Another domain-related matter is defining the role of risk-scoring using the RAM to drive the decision on tolerability of a particular risk. Multiple authors advise against using locations on a RAM (risk band) as the decision maker for tolerability of a hazard [8,11,12,13]. The concern about this is it extends the responsibility of risk-assessment team members to doing both the risk assessments (Process 3) and making decisions about tolerability (Process 4) without having all the information needed such as cost-benefit information.

### 1.3. Usability Issues

Members of a risk-assessment team will likely have differing opinions on assigning a hazard to a specific cell in their matrix. For that reason, RAMs should be designed to help the team members decide on the most appropriate row and column category. Three matrix attributes for helping risk-assessment team members make accurate and precise assignments to row and column categories are having: (i) a clear order to categories in each axis, (ii) descriptions of each category so that categories are distinguishable, and (iii) header terms that are clearly ordered and distinguishable. The third of these attributes has been the subject of only one previous study [21], and that was based on a survey of undergraduate OSH students. That left open an issue of how closely results of the undergraduate survey might correspond to ratings by individuals with OSH-related work experience.

Multiple usability issues involve the accuracy and precision of risk based on the judgment of risk-assessment teams. These estimates of risk are used by some organization to help set priorities for corrective actions A second use is to help decide if the risk-reduction tactics have reduced the risk of a hazard to the level of being tolerable or acceptable. Both uses are important to employee safety and health [9,11,12,13,14,15,16,17,18,19,20,21,22,23]. An example opinion expressed by Ale, Burnup, and Slater [9] is that using RAMs to prioritize risk-reduction processes may provide informative input, but should not be taken as a primary driver for prioritization. Similar opinions by other authors are that risk levels resulting from a risk-assessment team are not sufficiently accurate or precise to rely on as a sole determinant of risk tolerability [12,13]. Four implications of these opinions are that organizations need to make strong efforts to achieve accurate and precise entries into RAMs by (i) assigning competent individuals to risk-assessment teams, (ii) training risk-assessment team members for improving both accuracy and precision of assessments, (iii) providing team members with adequate time to do their assessments well, and (iv) adopting RAMs designed for usability.

The complexity of RAMs can contribute to usability. The form used in Figure 1 of this paper was based on both axes being linear and having equal ranges. Cox [13] presents justification for using that form of RAM for reasons including understandability, simplicity, and usability by risk assessors dealing with occupational hazards. He advises that three colored bands should be enough for RAMs designed for people estimating the row and column categories for a particular hazard. Cox also explained a rule to avoid having a green cell share an edge with a red cell. This reflects the reality that a risk-assessment team cannot be expected to reliably distinguish between adjacent categories of either scale. Having green and red cells share an edge invites misclassification errors, or what the human factors practitioners call design-induced errors.

The matrix format in Figure 3c has been discussed by numerous authors in papers about the spacing of categories [8,9,10,11,12,13,14]. A strength of this format is providing flexibility for a RAM designer to define the number of categories in each row and each column. While the common practice is to make equal width categories, unequal width categories may be used. For example, a five-category severity axis could be grouped so that the least harm category has the narrowest range while the greatest harm category has the widest range. Another example is setting the upper bounds of five likelihood categories at 1, 3, 5, 7, 10 [23]. Pons proposed simplifying required risk assessments by defining severity categories to align with those found in the applicable legislation [15].

Thus far in this article, the topic has been exclusively about two-dimensional risk matrices. These have been criticized for not including enough factors; in particular, the dimension of exposure is not included [11,21]. This concern may be addressed by either incorporating exposure into the likelihood dimension or adding a third dimension to account for extent of exposure. Terms for such a dimension were included in both the earlier study [21] and this follow-on study.

Another usability issue for RAM designers—selecting the terms for row and column headers—is an important attribute of RAMs that has received little attention. Duijm [11] commented that “the ways axis categories are defined and described” effects the subjective row and column category assignments. Baybutt [8] states that “different terms should not be used when the same meaning is intended”. He offered as an example naming adjacent severity categories with terms having essentially the same meaning, citing as examples significant injury and major injury. Duijm [11] pointed out the need to name categories on a single axis with clearly different descriptors and offered the following examples of misnaming adjacent categories by using terms that are listed as synonyms in a dictionary.

Improbable and seldom.
Often, frequent, and probable.
Disastrous and catastrophic.

Although Duijm’s examples were based on synonyms found in a dictionary, further support was subsequently provided by the survey of undergraduate OSH students reported by Jensen and Hansen [21]. They found that ratings on a 100-point likelihood scale were very close for the words improbable and seldom (mean 18.7 vs. 19.7 and median 20 vs. 18) as well as for frequent and probable (mean 72.0 vs. 68.2 and median 72.5 vs. 70.0). These authors also pointed out that MIL-STD-882E [22] uses the synonyms frequent and probable as labels for adjacent probability categories [21].

### 1.4. Reasons for a Second Survey

**Figure 4:**
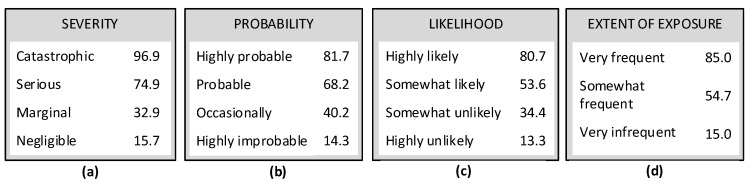
Example sets of terms based on means; adapted from prior study by Jensen and Hansen [21]: (**a**) set of four for severity, (**b**) set of four for probability, (**c**) set of four for likelihood, and (**d**) set of three for extent of exposure.

The previous recommendations were based on a survey completed by 84 undergraduate OSH students. The authors of that paper used the results to develop multiple sets of recommendations for RAMs of different sizes. Table 1 enumerates the number of categories and recommended word sets for each of the matrix axes studied. Examples of word sets are in Figure 4 along with mean ratings on a 100-point scale.

We undertook this survey with the aim of confirming or improving the prior recommended sets of terms [21] by using findings from a survey of people experienced in an OSH-related field and enrolled in an online graduate level course in industrial hygiene.

## 2. Materials and Methods

### 2.1. The Survey Instrument

An online survey was developed for this project. It asked respondents to rate various terms using a 100-point semantic differential scale available in the survey platform Qualtrics (Provo, Utah). It involved a linear rating scale with a mouse-controlled slide for indicating a rating from zero to 100. The end points were labeled with the bipolar descriptors below.

- For rating severity terms, the end points were No harm and Worst harm.
- For likelihood and probability terms, the end points were Impossible and Certain.
- For extent of exposure terms, the end points were No exposure and Constant exposure.

**Figure 5:**
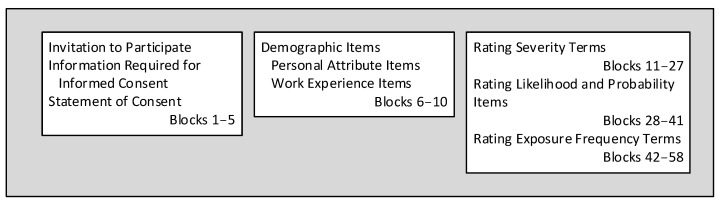
Organisation of survey instrument.

The survey instrument was designed to present sequential screens known as blocks. Figure 5 depicts how the blocks were arranged. Respondents were instructed to respond to a single item before advancing to another item. Respondents were not allowed to go backward to reconsider a term already rated.

Two surveys, identified as A and B, were created with identical material in Blocks 1 through 10. The terms rated were the same in both surveys with one unintended exception. One survey used minor harm, the other used minor damage. Within the categories (likelihood/probability, severity, and extent of exposure), the order of presentation was randomized for each survey. For example, the severity terms in Survey A were presented in random order, and the severity terms in Survey B were determined by a different random order.

The study was conducted according to the guidelines of the Declaration of Helsinki and approved by the Institutional Review Board of the University of Montana (protocol code 39-21, dated 21 February 2021). The approval was under the exempt category according to the U. S. Code of Federal Regulations, Part 42, section 104 (d).

### 2.2. Rationale for Terms Included in the Survey

The terms selected for this follow-on survey included a mix or identical terms, different terms, and some modified words. Table 2 lists the probability-based terms on the left and the likelihood terms on the right. Three probability-based terms were highly probable, probable, and improbable. The fourth term, remote, was in both surveys but, in the first survey, it was among the extent of exposure terms using a scale with end points No exposure and Constant exposure. In addition to remote, this second survey had six terms not previously studied. The term almost incredible was omitted from both lists for two reasons. One was that incredible means not credible and, according to Baybutt [8], events that are not credible should be excluded from risk analysis. Two, the prior study [21] found incredible had a very large standard deviation resulting from confusion among respondents as to whether it means near zero or near 100. In search of terms to replace almost incredible, we added extremely unlikely and extremely improbable to the second survey. In the prior survey, the lowest mean rating for a probability scale (14.3) was highly improbable. We sought an alternative term that would receive lower ratings, so we added extremely improbable, and, to mirror that on the high end of the rating scale, we added extremely probable.

Table 3 lists severity terms on the left and extent of exposure terms on the right. All severity terms were the same in both surveys with minor modifications. Among the extent of exposure terms in Table 3, a group of five were modified by adding “ly” to the end. A second group of four terms were modified by adding “exposed” to clarify that the intended meaning was how often exposure to the hazard occurred.

A third group of exposure terms consisted of four calendar-related terms (daily, weekly, monthly, annually). These were unchanged, because the authors of the earlier paper suggested that mixing these terms randomly within all the other extent of exposure terms might have influenced rating. In order to check this, the four terms were presented together as the final four rating items. Survey A and Survey B presented these four terms in different orders.

### 2.3. Procedures

An invitation to participate in a survey was extended to 98 individuals who were: (i) taking a Montana Technological University online course in industrial hygiene during spring semester 2021, (ii) engaged in a Master of Science program in industrial hygiene, and (iii) met the admission requirement of having at least two years of experience working in an occupational safety and health related job. In order to increase the response rate, the course instructors emailed their enrollees to watch for an invitation. None of the online courses were being taught by any of the researchers.

About two days after the notification emails, each student was sent a personal email invitation from the researchers to participate. The invitation did not contain any inducement to participate, such as points in their course grade, money, or other. Six or seven days after the invitation emails, the course instructors sent a second email to all their enrollees reminding them to consider participating if they had not already done so.

The 98 individuals were listed in a numbered order. Those with an odd number were sent a link to Survey A, while those with an even number were sent a link to Survey B. The individuals who chose to participate took the survey online. After starting the survey, respondents could stop at any point and their ratings were retained in the data set.

Analyses included reporting means, standard deviations, and medians for each term. Ratings for identical terms used in both surveys were compared using the Mann–Whitney test of medians [25]. The null hypothesis was the two data sets had equal medians while the alternate hypothesis was the two medians were not equal.

## 3. Results

### 3.1. Demographics of Respondents

The survey contained questions asking respondents for information about their personal attributes, most experience area of practice, and their present employment sector. For the personal attribute questions, items asked for first language, gender, and the ethnicity they most identify with. The age distribution, in decades, is provided in the left side of Table 4. The ages ranged from 26 to 60 with a mean of 38.9. For the question asking about language, 34 of 37 (91.1%) reported having English as their first language. For the three who reported other than English, their reported languages were Spanish, Chinese, and Yoruba.

When asked what ethnicity they identified with, the options were White/Caucasian, Hispanic/Latinx, Asian, Black/African-American, Native American/Native Alaskan, Hawaiian/Pacific Islander, and Other. One respondent provided no answer making a total of 36. A respondent who chose “Other” reported being African. No respondents chose Black/African American or Hawaiian/Pacific Islander. The numbers and percentages are listed in the right side of Table 4.

For their OSH-related work experience, the survey asked respondents for the practice area where they had the most experience. Responses are in the left side of Table 5. The first three experience areas listed in Table 5 are traditional categories of practice of occupational safety and health. These three accounted for 29 of the 37 (78.4%) respondents. Six others chose environmental protection. The survey category “Responder” was further defined in the survey to include emergency medical technicians, police, and firefighters. One respondent selected this area of practice.

The survey asked respondents about their current sector of employment. Results are in the right side of Table 5. The government category included Federal military (3) and Federal Non-Military (7). The latter consisted of six in other-than-public health and one in public health. The employment category Non-Federal Government had seven respondents, three employed in local (city/county) and four in state/provincial governments. The survey had options for healthcare and for environmental restoration that received zero responses. When asked about experience participating on a risk-assessment team, 27 of 37 (73.0%) reported having served on a risk-assessment team.

### 3.2. Ratings of Terms in Present Survey

Rating of the terms are in Table 6, Table 7, Table 8 and Table 9 for severity terms, probability terms, likelihood terms, and extent of exposure terms, respectively. All tables list the number of ratings (N), mean, standard deviation, and median. The order is according to the median. Where terms had equal medians, their order is according to mean rating.

Four terms were included in both Table 6 and Table 7 because these terms have meanings equally applicable to likelihood and probability. These terms were Certain, Almost Certain, Remote, and Fairly Normal.

Terms in each survey for extent of exposure are listed in Table 9 Four terms are expressed in terms of typical exposures (regularly exposed, occasionally exposed, seldom exposed, and rarely exposed). Five terms are for calendar-based exposures (daily, weekly, monthly, and annually). Four terms are for frequency-based exposures (very frequently, somewhat frequently, somewhat infrequently, infrequently, and very infrequently).

### 3.3. Parallel Wording

**Figure 6:**
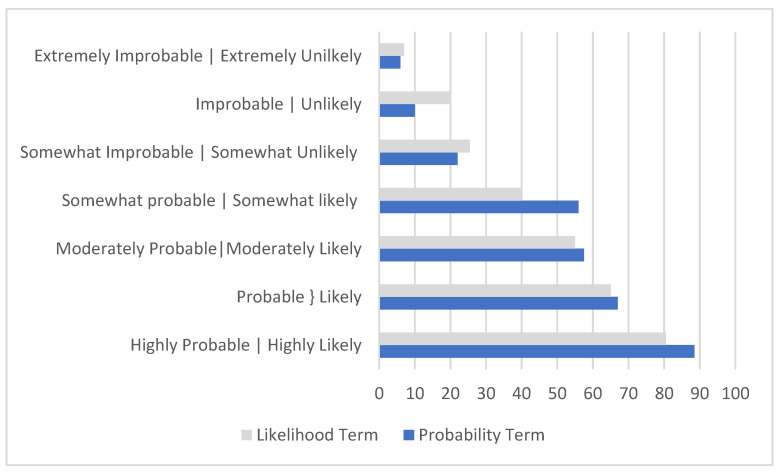
Bar chart comparing median ratings of likelihood and probability terms obtained in two surveys.

A consideration for selecting terms for likelihood and probability scales may include using one or more of the seven pairs of terms having parallel versions. All seven pairs of terms were rated using the same rating scale. The horizontal bar chart in Figure 6 provides a visual comparison, with the upper bar (gray) for the likelihood term and the lower bar (blue) for the comparable probability term. Four of the seven terms had closely matched medians.

Extremely improbable and extremely unlikely: (median 6, 7|mean 15.2, 15.9).
Somewhat improbable and somewhat unlikely: (median 22, 25.5|mean 28.5, 24.8).
Moderately probable and moderately likely: (median 57.5, 55|mean 61.1, 56.9).
Probable and likely: (median 67, 65|mean 65.3, 67.2).

The three parallel terms listed below had medians that were not as closely matched as the four above.

Highly probable and highly likely: (median 88.5, 81|mean 87.1, 84.2).
Improbable and unlikely: (median 10, 20|mean 14.1, 20.8).
Somewhat probable and somewhat likely: (median 56, 40|mean 57.2, 45.6).

### 3.4. Rating from Two Surveys Compared

Comparisons between median ratings from the undergraduates in the prior study [21] with ratings of corresponding terms in the present survey are provided in three tables—Table 10 for severity, Table 11 for probability and likelihood terms, and Table 12 for extent of exposure terms. Each table includes term-specific means, medians, difference in medians, and percentage difference, The Mann–Whitney test of medians identified different medians using the 0.05 level of significance (adjusted for ties) [25]. The order of terms in each table was based on difference in medians. For terms with equal differences, the order was based on largest to smallest *p*-value from the Mann–Whitney test. Each table presents term-specific means, medians, difference in medians, and percentage difference.

## 4. Discussion

This study was undertaken with the primary aim of confirming or improving the initial sets of terms [21] recommended for naming the rows and columns of risk assessment matrices by using findings from a survey of people experienced in an OSH-related field and enrolled in a graduate level course in industrial hygiene. Their recommendations were based on a survey of undergraduate OSH students. In contrast, this follow-on study was used to survey a sample of people with OSH-related experience. Based on findings of the follow-on survey, the authors (i) discuss their rationale for selectively removing some terms from further consideration due primarily to weak consistency between the two surveys (ii) considering calendar-based terms, and (iii) commenting on limitations of the investigation.

### 4.1. Selectively Removing Terms

A desirable attribute of terms to recommend for RAMs is consistency among different populations. For this study, a measure of consistency is the difference in medians between the prior and the present surveyed populations. Medians have an advantage over means by minimizing the contribution of outlier ratings. To help make decisions about retaining or removing terms, results of the two surveys were compared with a view toward consistency. Data in Table 10, Table 11 and Table 12 show results of comparing the two surveys. Although there is no natural difference in medians for separating those consistent versus inconsistent, after examining the comparison in those tables, the authors used judgment to sort terms into strong, moderate, and weak consistency, with the goal of removing those with weak consistency from recommendations.

Severity terms are in Table 10 along with term-specific differences in median (∆). Severity terms we classified as strongly consistent are: minor, catastrophic, minor damage, negligible, moderate, death of a person, serious, permanent injury/illness, severe, insignificant, and severe loss. These terms had differences in medians in the 0–5 range. Terms with moderate consistency were: critical and marginal with differences of nine and ten, respectively. Terms with weak consistency were: first aid only case (∆ = 13)*,* medical treatment case (∆ = 14)*,* and major damage (∆ = 16) with a difference greater than ten. We elected to remove the weak consistency terms for labeling the columns in a RAM. In addition, the terms major damage and minor damage were removed, however, if major damage is omitted, there is no need to retain minor damage, because it is redundant to the term minor as both have medians of 20.

Likelihood terms and probability terms used in both surveys are in Table 11. Terms we classified as strongly consistent were: certain, highly likely, unlikely, probable, likely and remote. These terms had differences in medians in the 0–5 range. Terms we classified as moderately consistent were: highly probable, somewhat unlikely, almost certain, and improbable. These terms had median differences in the 6–10 range. The only term in Table 11 considered weak in consistency, somewhat likely, had median ratings of 60 in the prior survey and 40 in the present survey (∆ = 20). This term was not preferred but was retained among terms to consider if no suitable alternative is identified.

### 4.2. Calendar-Based Terms

The four terms that express extent of exposure using calendar-based terms (daily, weekly, monthly, and annually) are appropriately considered as a group rather than being intermixed with other terms. The findings from the present survey show consistent spacing between these terms, specifically, the space between daily and weekly was 23.5, between weekly and monthly 26.5, and between monthly and annually 25. The authors of the prior paper [21] suggested that these terms might be rated differently if presented as a group, as was done in this survey. Table 13 provides comparative results. The difference supports consistency in order of medians and substantial consistency in median values. Differences between categories in the prior study were consistently 20 and 21. Those in the present survey were in the mid-twenties (23–27). It is concluded that these terms could be used to label a RAM with four categories and doing so would create acceptable spacing between categories.

### 4.3. Limitations

The survey described in this paper, and the prior survey, were based on target populations of people taking university courses. Because of that, we cannot generalize the findings to the diverse population of employed people who perform risk assessments in industry. For those actively involved in industrial risk assessment, their experience will have been influenced by their understanding of risk-related terminology. Moreover, because the risk-assessment terminology used in different industrial sectors is not uniform, we have no basis for expecting experienced risk assessors to have uniform or consistent understanding of the terms used in RAMs.

Another limitation is the number or respondents (*n* = 37). We have no way of knowing if those who responded are representative of the 98 invited to take the survey. What we do know is the 37 who responded are, as a group, more experienced in OSH-related jobs than the undergraduates who typically have an internship or no experience working in OSH. The findings that the two responder groups were, for the most part, consistent in their median rating of most terms adds confidence in the recommendations developed from the prior study.

## 5. Recommendations

Recommendations are presented in Table 14, Table 15, Table 16 and Table 17 for severity terms, likelihood terms, probability terms, and extent of exposure terms, respectively. Each table lists the recommended sets of terms from the survey of undergraduates [21], the mean the median of each term, the mean and median found in the present survey findings, and recommendations from the authors on each set. For severity sets in Table 14, findings from this follow-on survey are consistent with those of the prior survey [21], Two changes for consideration are: in the second set replace severe loss with severe, and in the third set replace major damage with severe loss.

For severity terms, nine of the 15 terms in Table 11 had median differences in the 0–5 range while six had large differences. Undergraduate rating of severity was higher than those of the graduate students for all difference over five. Three terms are not recommended: first aid cases (15.9), medical treatment cases (16.6), and major damage (12.9).

The ratings for likelihood terms in the prior and the present survey are presented in Table 15. Each of the sets included highly likely. It had similar ratings from both surveyed populations for means (80.7 and 84.2) and medians (80.5 and 81.0). The term somewhat likely appears to fill a gap in the middle range of likelihood. A concern about this term is the inconsistent rating between the prior survey and present survey, with means of 53.6 and 45.5 and medians of 60 and 40, respectively. In the set of three, there was no better term in these survey for naming the middle category of a likelihood axis in a RAM. The lowest term in the set of three (very unlikely) was among those recommended in the prior paper. A footnote indicates there are three terms suitable for the lowest category of a likelihood scale. The three terms with their medians are very unlikely (11),highly unlikely (10), and extremely unlikely (7). The research team suggests any of the three would be suitable. The sets of four and five in Table 16 have desirable spacing between them. The set of six, however, has two terms with minimal spacing, somewhat unlikely (25.5) and unlikely (20). The conclusion of the research team is that terms recommended in the prior paper are suitable for sets of three, four, and five. The set for six categories is sufficient, but not as well spaced as those in the other likelihood sets.

The ratings for probability terms in the prior and the present survey are presented in Table 16. The prior survey had only five probability terms (highly probable, probable, possible, improbable, and highly improbable). One consequence of that was lack of a probability term for the middle range. The prior authors decided to borrow the term occasionally from the extent of exposure terms. It had a mean rating of 40.2 using the extent of exposure rating scale. This was not an ideal solution. For the present survey, occasionally exposed was kept among the extent of exposure terms. In order to find terms to fill mid-range of the 100-point scale, the present survey included fairly normal, somewhat probable, and somewhat improbable. These terms are mentioned in the Recommendations column of Table 16.

The primary conclusion of the research team is that probability terms recommended in the prior paper had insufficient options for creating categories with appropriate spacing. The rational for improvements are provided in Table 16.

The ratings for extent of exposure terms in the prior and the present survey are presented in Table 17. Minimal modifications to the prior recommended terms were made before conducting the present survey. One such modification was adding the word ”exposed” after regularly, seldom, occasionally, and rarely. The reason was to help survey respondents think about how the term is to be used. The other modification was to add “ly” to the words frequent and infrequent. Other than those changes, the prior sets of terms were confirmed and supported by findings from the present study. The set of two would be suitable as a third axis in a RAM. It could be operationalized as two traditional RAMs set side by side, one for regularly exposed and one for seldom exposed. The sets of three could also be operationalized in that way as well. The present authors agree with the prior authors that extent of exposure is best regarded as a set of only two or three categories.

Findings for severity indicated a few terms that should not be used for naming the rows and columns of risk assessment matrices. Do not use first aid case only or medical treatment case because ratings of these terms appear to be influenced by reporting requirement and workers’ compensation laws. These terms would fit better in the text descriptions of the severity categories.

Findings for likelihood indicated the adjectives “very” and “extremely” have similar meanings when used to modify likely and probable. Therefore, using one of these but not both is recommended. Some adjectives produced similar effects when used to modify the terms likely and probable. Extremely improbable and extremely unlikely produce ratings of 6 and 7. Moderately probable and moderately likely received median ratings of 67 and 65. Somewhat improbable and somewhat unlikely received median ratings of 22 and 25.5. Highly probable and highly likely had median ratings of 88.5 and 80.5. The bar chart in Figure 6 facilitates comparison.

## 6. Conclusions

The aim of this project was to confirm or improve the prior recommended word sets for headers of the columns and rows in RAMs. Findings led to the following conclusions.

1. The survey confirmed the prior recommendations for severity terms. However, the authors recommend limiting use of the set containing the word “damage” to hazards concerned with harm to equipment, facilities, products and the environment.
2. The survey confirmed the prior recommendations for likelihood terms with some suggestions. The term somewhat likely had a median in this survey of 40, but a median of 60 in the prior survey. That does not negate use of the term, but due to the inconsistent ratings, we suggest using moderately likely with a median rating of 55.
3. Based on ratings in both surveys, the ratings for the terms for the lowest likelihood category did not produce a winner. Three terms intended for naming the lowest category, with their medians, are: very unlikely (11), extremely unlikely (7), or highly unlikely (10). We express no preference.
4. The survey found concerns with some terms in the probability sets. The prior survey did not include terms with rating in the middle range of probability, so four terms were added to the survey: fairly normal, moderately, somewhat probable, and somewhat improbable. Rating for these terms provides alternatives for the word occasionally in the sets found in Table 16. The authors recommend replacing occasionally in the upper set with fairly normal, and in the three lower sets with somewhat improbable.
5. The survey confirmed the prior recommendations for extent of exposure with small changes. An improvement incorporated into the present survey was adding the word “exposed” to four words in the prior survey to make four terms—regularly exposed, occasionally exposed, seldom exposed, and rarely exposed.

## Figures and Tables

**Table 1:** Word sets recommended are adapted from the paper by Jensen and Hansen [21] and organized in this table by number of categories in the axis.

Axis Parameter	Number of Categories	Number of Sets Recommended	Example
Severity	Three	3	
	Four	1	Figure 4a
	Five	2	
Probability	Three	1	
	Four	1	Figure 4b
	Five	1	
	Six	1	
Likelihood	Three	1	
	Four	1	Figure 4c
	Five	1	
	Six	1	
Extent of Exposure	Two	1	
	Three	2	Figure 4d

**Table 2:** Probability-based terms and likelihood-based terms in the survey and whether the terms are the same or different from the prior survey by Jensen and Hansen [21].

Probability Terms			Likelihood Terms		
Term Studied	Same	Different	Term Studied	Same	Different
Highly probable	X		Highly likely	X	
Probable	X		Likely	X	
Improbable	X		Somewhat likely	X	
Remote		X	Somewhat unlikely	X	
Fairly normal		X	Unlikely	X	
Moderately probable		X	Certain	X	
Extremely probable		X	Almost certain	X	
Extremely improbable		X	Extremely unlikely		X
Somewhat probable		X	Extremely likely		X
Somewhat improbable		X	Moderately likely		X
			Fairly normal		X
			Very unlikely		X
			Very likely		X

**Table 3:** Terms in the survey for severity of harm and extent of exposure along with indicating if same or changed from prior survey by Jensen and Hansen [21].

Severity Terms		Extent of Exposure Terms	
Current Study Term	Prior Study	Current Study Terms	Prior Study Terms
Catastrophic	Same	Very frequently	Very frequent
Medical treatment case	Same	Frequently	Frequent
Severe	Same	Somewhat frequently	Somewhat frequent
Moderate	Same	Infrequently	Infrequent
Minor damage	Same	Very infrequently	Very infrequent
Insignificant	Same	—	—
Serious	Same	Regularly exposed	Regularly
Severe loss	Same	Occasionally exposed	Occasionally
Major damage	Same	Seldom exposed	Seldom
Negligible	Same	Rarely exposed	Rarely
Permanent injury/illness	Same	—	—
Critical	Same	Annually	Same
Minor	Same	Monthly	Same
Death of a person ^1^	Same	Weekly	Same
First aid only case	Same	Daily	Same
Marginal	Same		

^1^ Prior survey used the term “Death of one person”.

**Table 4:** Attributes of respondents.

Age	N	Prct.	Gender	N	Prct.	Ethnicity or Race	N	Prct.
60–69	1	02.7	Male	25	69.4	White/Caucasian	27	75.0
50–59	5	13.5	Female	11	30.6	Hispanic/Latinx	4	11.1
40–49	11	29.7	Decline	1	NA	Asian	3	8.3
30–39	12	32.4				Native American ^1^	1	2.8
20–29	8	21.6				Other (African)	1	2.8
Total	37	99.9 ^2^		37	100.0		36	100.0

^1^ The category included native Americans and native Alaskans. ^2^ Not precisely 100.0 due to rounding.

**Table 5:** Most experience area of practice and current employment sector.

Most Experience	N	Prct.	Sector Employed	N	Prct.
Occupational Safety	5	13.5	Private Industrial	9	25.0
Industrial Hygiene	12	32.4	Private Commercial	5	13.9
Occupational S&H Combined	12	32.4	Education	4	11.1
Environmental Protection	6	16.2	Federal Military	3	8.3
Responder	1	2.7	Federal Non-Military	7	19.4
Other (not specified)	1	2.7	Non-Federal Government	7	19.4
			Other	1	2.8
Total	37	99.9 ^1^	Total	36	99.9 ^1^

^1^ Not precisely 100.0 due to rounding.

**Table 6:** Ratings of severity terms ordered by median.

Term Rated	N	Mean	St. Dev.	Median
Death of a person ^1^	32	99.7	1.4	100.0
Catastrophic	33	96.4	6.5	100.0
Permanent Injury/Illness	33	87.3	18.9	92.0
Severe Loss	34	77.1	11.8	85.0
Critical	34	78.9	13.4	81.0
Severe	34	77.1	11.8	80.0
Serious	34	71.0	13.8	70.0
Major Damage	30	71.3	17.0	70.5
Medical Treatment Case	34	57.4	19.1	60.0
Moderate	34	44.9	12.6	50.0
First Aid Only Case	34	25.9	16.4	24.5
Marginal	33	26.4	12.8	21.0
Minor Damage	33	22.9	8.7	20.0
Minor	33	20.6	10.1	20.0
Negligible	29	21.3	26.0	10.0
Insignificant	26	10.5	16.5	5.5

^1^ All ratings for Death of a person were 100 or 99 except one extreme outlier of 3 was removed from the data set prior to analyses.

**Table 7:** Ratings for probability terms ordered by median.

Term Rated	N	Mean	St. Dev.	Median
Certain	31	95.1	12.4	100.0
Extremely Probable	31	93.9	4.9	95.0
Almost Certain	33	92.1	7.0	94.0
Highly Probable	30	87.8	7.9	88.5
Probable	31	65.4	16.8	67.0
Moderately Probable	30	61.1	14.2	57.5
Somewhat Probable	28	57.3	15.0	56.0
Fairly Normal	31	53.5	22.7	51.0
Somewhat Improbable	31	28.5	14.2	22.0
Remote	30	25.1	22.4	16.5
Improbable	27	14.1	9.3	10.0
Extremely Improbable	25	15.2	24.9	6.0

**Table 8:** Ratings for likelihood terms ordered by median.

Term Rated	N	Mean	St. Dev.	Median
Certain	31	95.1	12.4	100.0
Almost Certain	33	92.1	7.0	94.0
Extremely Likely	30	87.0	16.4	90.0
Highly Likely	31	84.2	9.4	81.0
Likely	31	67.2	16.9	65.0
Moderately Likely	31	56.9	12.5	55.0
Fairly Normal	31	53.5	22.7	51.0
Somewhat Likely	31	45.5	16.4	40.0
Somewhat Unlikely	32	24.8	13.0	25.5
Unlikely	27	20.8	12.3	20.0
Remote	30	25.1	22.4	16.5
Extremely Unlikely	28	15.9	26.7	7.0

**Table 9:** Ratings of extent of exposure ordered by median.

Term Rated	N	Mean	St. Dev.	Median
Daily ^1^	30	90.1	12.2	94.0
Very Frequently	31	80.8	17.0	82.0
Regularly Exposed	30	75.1	18.7	77.5
Frequently	31	72.7	16.2	75.0
Weekly ^1^	30	64.6	21.4	70.5
Somewhat Frequently	31	56.7	16.2	60.0
Monthly ^1^	31	42.4	18.3	44.0
Occasionally exposed	31	39.2	24.8	31.0
Somewhat Infrequently	31	30.1	17.6	27.0
Infrequently	30	22.2	16.1	20.5
Annually ^1^	30	21.9	17.7	19.0
Seldom Exposed	31	17.4	19.3	11.0
Very Infrequently	29	14.7	21.0	10.0
Rarely Exposed	25	9.6	11.4	6.0

^1^ Calendar-based Terms.

**Table 10:** Ratings for severity terms from the prior survey of undergraduates by Jensen and Hansen [21] compared to present survey of experienced graduate students, ordered by difference (∆) in median rating.

Terms for Severity of Harm	Previous Survey: Undergraduates		Present Survey: Experienced		∆ Medians ^1^	% Diff ^2^
	Mean	Median	Mean	Median		
Minor	21.8	20	20.6	20	0.0	0.0
Catastrophic	96.8	100	96.4	100	0.0	0.0
Minor damage	25.0	20	22.3	20	0.0	0.0
Negligible	15.7	10	21.3	10	0.0	0.0
Moderate	48.9	50	44.9	50	0.0	0.0
Death of one person ^3^	97.1	100	99.8	100	0.0	0.0 *
Serious	74.9	74	71.0	70	4.0	−5.4
Permanent Injury/illness	94.4	96	87.3	92	4.0	−4.2
Severe	83.8	84	77.1	80	4.0	−4.8 *
Insignificant	12.6	10	10.5	5.5	4.5	−45.0 *
Severe loss	86.9	90	85.1	85	5.5	−5.6 *
Critical	84.5	90	78.9	81	9.0	−10.0 *
Marginal	32.9	31	24.8	21	10.0	−32.3 *
First aid only case	41.8	37.5	25.9	24.5	13.0	−34.7 *
Medical treatment case	74.0	74	57.4	60	14.0	−18.9 *
Major damage	81.7	86	71.3	70.5	15.5	−18.0 *

^1^ Previous survey median minus present survey median. ^2^ Percent difference = 100 ((Median1 − Median2)/Median1). ^3^ Present survey used “Death of a person” whereas prior survey used “Death of one person”. This may be the reason the difference tested significant. * indicates significant difference at *p* < 0.05 according to Mann–Whitney test of medians.

**Table 11:** Ratings for likelihood and probability terms from the prior survey of undergraduates by Jensen and Hansen [21] compared to present survey of experienced graduate students, ordered by difference (∆) in median rating.

Terms for Likelihood and Probability	Previous Survey: Undergraduates		Present Survey: Experienced		∆ Medians ^1^	% Diff ^2^
	Mean	Median	Mean	Median		
Certain	94.9	100.0	95.1	100.0	0.0	0.0
Highly Likely	80.7	80.5	84.2	81.0	−0.5	−0.6
Unlikely	24.9	21.0	20.8	20.0	1.0	4.8
Probable	67.4	70.0	65.4	67.0	3.0	4.3
Likely	65.2	70.0	67.2	65.0	5.0	4.8
Highly Probable	81.7	82.0	87.8	88.5	−6.5	−7.9 *
Somewhat Unlikely	34.4	34.0	24.8	25.5	8.5	25.0 *
Almost Certain	81.4	85.0	92.1	94.0	−9.0	−10.6 *
Improbable	18.7	20.0	14.1	10.0	10.0	50.0 *
Somewhat Likely	53.4	60.0	45.5	40.0	20.0	33.3 *

^1^ Previous survey median minus present survey median. ^2^ Percent difference = 100 ((Median1 − Median2)/Median1). * indicates significant difference at *p* < 0.05 according to Mann–Whitney test of medians.

**Table 12:** Ratings for extent of exposure terms from the prior survey of undergraduates by Jensen and Hansen [21] compared to present survey of experienced graduate students, ordered by difference (∆) in median rating.

Term for Extent of Exposure	Previous Survey: Undergraduates		Present Survey: Experienced		∆ Medians ^1^	% Diff ^2^
	Mean	Median	Mean	Median		
Very Infrequently	15.0	10.0	14.7	10.0	0.0	0.0
Infrequently	23.1	20.0	22.2	20.5	−0.5	−2.5
Weekly	65.9	70.0	62.5	70.5	−0.5	−0.7
Somewhat Frequently	54.0	59.5	56.7	60.0	−0.5	−0.8
Regularly Exposed	74.1	74.0	75.1	77.5	−3.5	−4.7
Frequently	72.0	72.5	72.7	75.0	−2.5	−3.4
Remote ^3^	16.7	14.0	25.1	16.5	−2.5	−17.9
Daily	86.8	90.0	90.1	94.0	−4.0	−4.4
Occasionally exposed	39.6	36.0	39.2	31.0	5.0	13.9
Monthly	49.3	50.0	42.4	44.0	6.0	12.0
Very Frequently	85.0	88.5	80.8	82.0	6.5	7.3
Seldom Exposed	19.7	18.0	17.4	11.0	7.0	38.9 *
Rarely Exposed	15.6	14.0	9.6	6.0	8.0	57.1 *
Annually	36.2	29.5	21.9	19.0	10.5	35.6 *

^1^ Previous survey median minus present survey median. ^2^ Percent difference = 100 ((Median1 − Median2)/Median1). ^3^ Remote rated on likelihood scale in previous survey, but on extent of exposure scale in present survey. * indicates significant difference at *p* < 0.05 according to Mann–Whitney test of medians.

**Table 13:** Comparison of median ratings from the prior survey by Jensen and Hansen [21] and this follow-on survey for calendar-based terms.

Term	Prior Survey Median	Present Survey Median	Difference
Daily	90.0	94.0	−4.0
Weekly	70.0	70.5	0.0
Monthly	50.0	44.0	6.0
Annually	29.5	19.0	10.5

**Table 14:** Sets of three, four, and five terms for severity as recommended in prior paper [21] compared to present survey with comments by the research team. Prior survey data adapted from Jensen and Hansen [21].

Sets of Terms from Prior Survey	Prior Survey		Survey of Graduates		Recommendations
	Mean	Median	Mean	Median	
Severe	83.8	84	77.1	85.0	Recommended with no change
Moderate	48.9	50	44.9	50.0	
Minor	21.8	20	20.6	20.0	
Severe loss	86.9	85	77.1	85.0	Recommended but replace severe loss with severe
Moderate	48.9	50	44.9	50.0	
Minor	21.8	20	20.6	20.0	
Major damage	81.9	86	71.3	70.5	Recommended for equipment, facilities, environment but not for human safety and health.
Moderate	48.9	50	44.9	50.0	
Minor damage	25.6	20	22.9	20.0	
Catastrophic	96.9	100	96.4	100.0	Recommended with no change
Serious	74.9	74	71.0	70.0	
Marginal	32.9	31	26.4	21.0	
Negligible	15.7	10	21.3	10.0	
Catastrophic	96.9	100	96.4	100.0	Recommended with no change
Severe	83.3	84	77.1	80.0	
Moderate	48.9	50	44.9	50.0	
Marginal	32.9	31	26.4	21.0	
Insignificant	12.6	10	10.5	5.5	
Catastrophic	96.9	100	96.4	100.0	Recommended with no changes
Serious	74.9	74	71.0	70.0	
Moderate	48.9	50	44.9	50.0	
Marginal	32.9	31	26.4	21.0	
Insignificant	12.6	10	10.5	5.5	

**Table 15:** Sets of three, four, five and six terms for likelihood recommended in prior paper [21] compared to present survey with recommendations by the research team. Prior survey data adapted from Jensen and Hansen [21].

Sets of Terms from Prior Survey [18]	Prior Survey		Survey of Graduates		Recommendations
	Mean	Median	Mean	Median	
Highly likely	80.7	80.5	84.2	81.0	Recommended with options to consider in footnotes 1 and 2
Somewhat likely ^1^	53.6	60.0	45.5	40.0	
Very unlikely ^2^	14.6	11.0	No match	No match	
Highly likely	80.7	80.5	84.2	81.0	Recommended with options to consider in footnotes 1 and 2
Somewhat likely ^1^	53.6	60.0	45.5	40.0	
Somewhat unlikely	34.4	34.0	24.8	25.5	
Highly unlikely ^2^	13.3	10.0	No match	No match	
Certain	96.0	100	95.1	100.0	Recommended with options to consider in footnotes 1 and 2
Highly likely	80.7	80.5	84.2	81.0	
Somewhat likely ^1^	53.6	60.0	45.5	40.0	
Somewhat unlikely	34.4	34.0	24.8	25.5	
Highly unlikely ^2^	13.3	10.0	No match	No match	
Highly likely	80.7	80.5	84.2	81.0	Recommended with options to consider in footnotes 1 and 2
Likely	66.0	70.0	67.2	65.0	
Somewhat likely ^1^	53.6	60.0	45.5	40.0	
Somewhat unlikely	34.4	34.0	24.8	25.5	
Unlikely	24.6	22.0	20.8	20.0	
Highly unlikely ^2^	13.3	10.0	No match	No match	

^1^ A concern with the term somewhat likely is it had inconsistent ratings from the two survey populations (medians of 60 and 40). If an alternative is desired, the term moderately likely (mean 56.9, median 55) would be suitable. ^2^ A term for the lowest likelihood category in a RAM could be any of three: very unlikely (11), extremely unlikely (7), or highly unlikely (10). Median ratings are in parentheses. The authors see no clear preference.

**Table 16:** Sets of three, four, five, and six terms for probability recommended in prior paper [21] compared to present survey with comments by the research team. Prior survey data adapted from Jensen and Hansen [21].

Sets of Terms from Prior Survey	Prior Survey		Survey of Graduates		Recommendations
	Mean	Median	Mean	Median	
Highly probable	81.7	82	87.8	88.5	Recommended with options to consider footnotes 1 and 2
Occasionally ^1^	40.2	36	No match	No match	
Highly improbable ^2^	14.3	10	No match	No match	
Highly probable	81.7	82	87.8	88.5	Recommend with options to consider in footnotes 1 and 2.
Probable	68.2	70	65.4	67.0	
Occasionally ^1^	40.2	36	No match	No match	
Highly improbable ^2^	14.3	10	No matcch	No match	
Highly probable	81.7	82	87.8	88.5	Recommend with comments: Replace possible with somewhat probable (mean 57.3, median 56). Replace occasionally with somewhat improbable (mean 28.5, median 22).
Probable	68.2	70	65.4	67.0	
Possible	59.4	60	No match	No match	
Occasionally ^1^	40.2	36	No match	No match	
Highly improbable ^2^	14.3	10	No match	No match	
Certain	96.0	100	95.1	100.0	Recommend with options to consider in footnotes 1 and 2
Highly probable	81.7	82	87.8	88.5	
Probable	68.2	70	65.4	67.0	
Possible	59.4	60	No match	No match	
Occasionally ^1^	40.2	36	No match	No match	
Highly improbable ^2^	14.3	10	No match	No match	

^1^ The term occasionally is a better fit for extent of exposure than it is for probability. For the probability sets, the authors recommend somewhat improbable with median 22. ^2^ The term highly improbable had a median of 10 in the prior survey. If an alternative is desired, either improbable (10) or extremely improbable (6) would be suitable.

**Table 17:** Sets of two and three terms for extent of exposure recommended in prior paper [21] compared to present survey with recommendations by the present research team. Prior survey data adapted from Jensen and Hansen [21].

Sets of Terms from Prior Survey	Prior Survey		Survey of Graduates		Recommendations
	Mean	Median	Mean	Median	
Regularly ^1^	74.1	74.0	75.1	77.5	Recommended with minor word change ^1^
Seldom ^1^	19.7	18.0	17.4	11.0	
Regularly ^1^	74.1	74.0	75.1	77.5	Recommended with minor word change ^1^
Occasionally ^1^	40.2	36.0	39.2	31.0	
Rarely ^1^	15.8	14.0	9.6	6.0	
Very frequent ^2^	85.0	88.5	80.8	82.0	Recommended with minor word change ^2^
Somewhat frequent ^2^	54.7	59.5	56.7	60.0	
Very infrequent ^2^	15.0	10.0	14.7	10.0	

^1^ Added in present survey “exposed” after Regularly, Seldom, Occasionally, and Rarely. ^2^ Added in present survey “ly” to the words frequent and infrequent.

**Figure A1:**
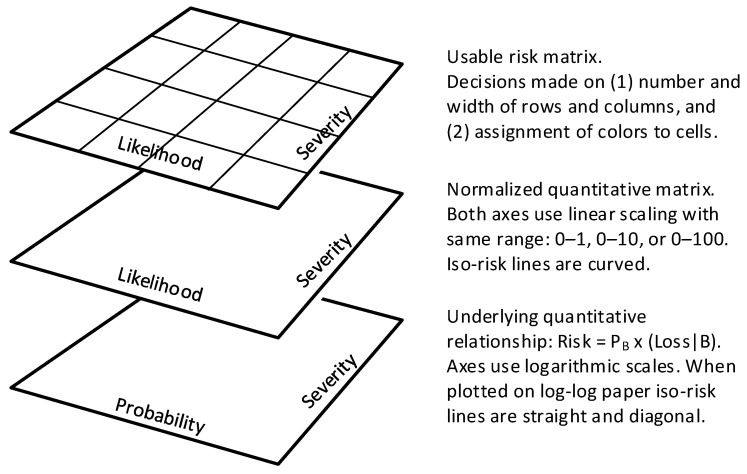
A 3-floor building analogy depiction of how an underlying quantitative relationship using logarithmic scaling (ground floor) may be normalized to form a quantitative matrix using linear scaling (middle floor). The top floor is carpeted using rectangular pieces of carpeting colored red, yellow, and green, arranged in a pattern to identify spaces of similar risk.

## Data Availability

The following are available online at Tables S1–S3.

## Supplementary Materials

The following are available online at https://www.mdpi.com/article/10.3390/ijerph19052763/s1, Table S1: Ratings of Probability and Likelihood Terms, Table S2: Ratings of Exposure Terms, Table S3: Ratings of Severity Terms.

## Appendix A. Rationale for Normalized, Equal-Axis Risk Matrices

The quantitative risk matrix used in the system safety profession has rows with numerical values for probability defined to fit the situation of concern. The columns have numerical values of consequence, commonly in expected dollar value of loss, or, in some domains, consequence may be in number of lives lost. In the practice of occupational safety and health, the values required for both probability and severity are imprecise estimates made by people. For example, should the probability of a particular hazardous event be 10^−3^ or 10^−6^? What amount should be used for the death of one employee? What is needed for OSH is a RAM formatted to accommodate human estimates of both axes.

The RAM in Figure 1 of the main article is based on a framework with both axes having a 0–10 range, and the whole space divided into cells based on the intersection of row and columns. Tony Cox explained the mathematical and statistical rationale in a 2008 paper [13]. An attempt to explain the rationale in a less rigorous manner follows.

Figure A1 depicts three planes analogous to a three-floor building. The ground floor represents the underlying quantitative relationship between probability and severity as an X-Y graph. A plot of the X-Y space on log-log paper can be used to plot lines of equal risk using Equation (1). These iso-risk lines run straight from the upper left toward the lower right.

The next floor up is based on changing the logarithmic axis scales into linear scales by normalizing each to a specified range. The linear range of each axis described by Cox was 0–1. Equivalent scales may use 0–10 or 0–100. On this floor, bands of similar risk are defined by curved iso-risk lines plotted in this X-Y space like those shown in Figure 1 of the main article. For example, the iso-risk line at 45 in Figure 1 defines a space above and right of the line as a high-risk region, and the iso-risk line 20 defines the space to its left and below as the low-risk region. This is all good technically, but a typical risk assessment team in industry using this approach needs to reach agreement on numerical values for both axes in order to determine the point in the X-Y space where a particular hazard belongs. This could take a lot of time and possibly lead to bickering among the team members. For that reason, a RAM format that is more accommodating for human judgment is desirable.

The upper floor in the building represents the usable risk matrix for assessing hazards. It uses the same axes as the floor below, including the iso-risk lines. The building owner may retain a RAM designer to install rectangular pieces of colored carpet to lay in a grid pattern. If carpet colors are red, yellow, and green, the pattern could mirror the layout in Figure 1, or a different pattern preferred by the building owner or RAM designer.
